# Bridging mouse and human anatomies; a knowledge-based approach to comparative anatomy for disease model phenotyping

**DOI:** 10.1007/s00335-023-10005-4

**Published:** 2023-07-08

**Authors:** Jesús Ruberte, Paul N. Schofield, John P. Sundberg, Alfonso Rodriguez-Baeza, Ana Carretero, Colin McKerlie

**Affiliations:** 1 Center for Animal Biotechnology and Gene Therapy, Universitat Autònoma de Barcelona, Barcelona, Spain; 2 Department of Animal Health and Anatomy, Universitat Autònoma de Barcelona, Barcelona, Spain; 3 The Jackson Laboratory, Bar Harbor, ME USA; 4 Department of Physiology, Development and Neuroscience, University of Cambridge, Cambridge, UK; 5 Department of Dermatology, Vanderbilt University Medical Center, Nashville, TN USA; 6 Department of Morphological Sciences, Universitat Autònoma de Barcelona, Barcelona, Spain; 7 The Hospital for Sick Children, Toronto, Canada; 8 Department of Lab Medicine and Pathobiology, Faculty of Medicine, University of Toronto, Toronto, Canada

## Abstract

The laboratory mouse is the foremost mammalian model used for studying human diseases and is closely anatomically related to humans. Whilst knowledge about human anatomy has been collected throughout the history of mankind, the first comprehensive study of the mouse anatomy was published less than 60 years ago. This has been followed by the more recent publication of several books and resources on mouse anatomy. Nevertheless, to date, our understanding and knowledge of mouse anatomy is far from being at the same level as that of humans. In addition, the alignment between current mouse and human anatomy nomenclatures is far from being as developed as those existing between other species, such as domestic animals and humans. To close this gap, more in depth mouse anatomical research is needed and it will be necessary to extent and refine the current vocabulary of mouse anatomical terms.

## Introduction

The mouse remains the key animal model for exploring human disease and, despite its small comparative size, the laboratory mouse is anatomically similar to humans, providing even unexpected anatomical analogies in structures with high interspecies variation such as the presence of the clavicle. Knowledge of human anatomy has been collected and recorded throughout human history, however, the first comprehensive study of mouse anatomy was published by Margaret J. Cook in 1965 (Cook 1965). Since the release of Cook’s atlas, several books and resources of mouse anatomy and have been published (Table 1). Nevertheless, the understanding and published descriptions of mouse anatomy (*Mus musculus*) is far from the body of knowledge available for humans (*Homo sapiens*). The most recently discovered anatomical structure in humans, the cisterna chyli, was first described four hundred years ago (Natale et al. 2017). In contrast, there still remain examples of anatomical structures of the mouse such as the bulbourethral glands with only poorly described structural detail. Other examples of the gap in complete description between human and mouse anatomy are: (1) the extant knowledge of normal human anatomical variation is well documented and quantified, but is poorly characterized in the mouse. As an example, a previously accepted anatomical difference between mouse and human, the presence of a supratrochlear foramen in the humerus, is now known to be incorrect because a percentage of humans present with this foramen; (2) the disparity between the number of anatomical entities in “gold-standard” ontologies: 3257 in the Mouse Anatomy (MA) ontology (version 2017-02-07) (Hayamizu et al. 2005b) versus 7800 in the Terminologia Anatomica (TA) (version 2021-16-08) (F.I.P.A.T 2019); and (3) existing mouse and human anatomic ontologies (Rosse and Mejino 2008) have only ~ 50% of terms have a conceptual or lexical match.

**Table 1:** Publications on mouse anatomy

Title	Year	Authors	Publisher	Characteristics
Biology of the Laboratory Mouse	1941	Snell GD	Dover Publications	One chapter on mouse anatomy by Fekete E. Drawings and photographs from histological sections
The Anatomy of the Laboratory Mouse	1965	Cook MJ	Academic Press	Atlas of drawings. Freely available at: http://www.informatics.jax.org/cookbook
Atlas of the Mouse Brain and Spinal Cord	1971	Sidman RL, Angevine JB, Pierce ET	Harvard University Press	Atlas with coronal, sagittal, and horizontal brain and spinal sections and diagrams. Nomenclature in Latin
The Mouse in Biomedical Research	1983	Foster HL, Small JD, Fox JG	Academic Press	One chapter on mouse anatomy by MJ Cook
Necropsy Guide: Rodents and the Rabbit	1988	Feldman DB, Seely JC	CRC Press	Anatomical basis for the mouse necropsy. Photographs and text
A Guided Tour of Veterinary Anatomy. Domestic Ungulates and Laboratory Mammals	1992	Smallwood JE	WB Saunders Company	One chapter dedicated to laboratory mammal anatomy. Text and diagrams
Colour Atlas of Anatomy of Small Laboratory Animals. Volume two: Rat, Mouse, and Hamster	1992	Popesko P, Rajtová V, Horák J	Saunders Company	Atlas of colour drawings. Uses Nomina Anatomica Veterinaria in Latin and English
Pathology of the Mouse	1999	Maronpot RR	Cache River Press	Anatomical and histological introduction in each of the chapters
Systematic Evaluation of the Mouse Eye. Anatomy, Pathology, and Biomethods	2000	Smith RS, John SWM, Nishina PM, Sundberg JP	CRC Press	One section (3 chapters) dedicated to the eye anatomy and development. Text and photographs
Atlas of Mouse Hematopathology	2000	Fredrickson TN and Harris AW	Harwood Academic	Three chapters about the anatomy, histology, and cytology of lymphatic and hematopoietic systems
Pathology of Genetically Engineered Mice	2000	Ward JM, Mahler JF, Maronpot RR, Sundberg J, Frederickson RM	Iowa State Press	Several chapters with anatomical introductions
A color Atlas of Sectional Anatomy of the Mouse	2001	Iwaki T, Yamashita H, Hayakawa T	Braintree Scientific Inc	Color atlas with photographs of skeleton, dissections, and transverse, sagittal, and dorsal sections
Laboratory Animal Medicine. Second Edition	2002	Fox JG, Anderson LC, Loew FM, Quimby, FW	Academic Press	Short introduction to the mouse anatomy taken from Cook’s book
Atlas of Laboratory Mouse Histology	2004	Conti CJ, Gimenez-Conti IB, Benavides F, Frijhoff AFW, Conti MA	Texas Histopages Inc	Atlas with photographs and diagrams. CD format
Anatomia degli Animali da Laboratorio	2006	Cozzi B, Ballarin C, Peruffo A, Carù F	Casa Editrice Ambrosiana	Atlas with photographs from dissections and histological sections
The Mouse Brain in Stereotaxic Coordinates. Compact Third Edition	2008	Paxinos G and Franklin KBJ	Academic Press	Atlas with 100 coronal histological brain sections (photographs and diagrams)
Allen Reference Atlas. A Digital Color Brain Atlas of the C57BL/6J Male Mouse	2008	Dong HW	Wiley	Atlas with 151 histological brain sections (coronal and sagittal) accompanied by diagrams and a systematic hierarchically organized taxonomy. https://mouse.brain-map.org
Histologic Basis of Mouse Endocrine System Development: A Comparative Analysis	2010	Kaufman M, Nikitin AY, Sundberg JP	CRC Press	Anatomy, histology and development of ovary, testis, pancreas, hypophysis, thyroid, parathyroid, and pineal glands
Comparative Anatomy of the Mouse and the Rat	2011	Constantinescu GM	CRC Press	Color atlas with illustrations drawn from dissections. Uses the Nomina Anatomica Veterinaria
The Mouse Nervous System	2011	Watson C, Paxinos G, Puelles L	Academic Press	Text with photographs providing a comprehensive anatomy of the central nervous system of the mouse
Comparative Anatomy and Histology. A Mouse and Human Atlas	2012	Treuting PM, Dintzis SM, Frevert CW, Liggitt D	Academic Press	Text and Atlas. For comparison with human anatomy uses Netter images and MA ontology
The Laboratory Mouse. Second edition	2012	Hedrich HJ	Academic Press	One chapter on mouse anatomy by Komárek V. Drawings taken from Popesko et al. (2002)
A Practical Guide to the Histology of the Mouse	2014	Scudamore CL	Willey Blackwell	Histological text and atlas with the basis of mouse anatomy
Morphological Mouse Phenotyping. Anatomy, Histology and Imaging	2016 2017	Ruberte J, Carretero A, Navarro M	Editorial Medica Panamerica Academic Press	Text and atlas. 2200 original photographs from dissections, histological sections, and imaging. Uses the Nomina Anatomica Veterinaria
Comparative Anatomy and Histology. A Mouse, Rat, and Human Atlas	2018	Treuting PM, Dintzis SM, Montine KS	Academic Press	Text and Atlas. This second edition adds the rat as comparator species
The Laboratory Mouse. A guide to the Location of Tissues for Optimal Histological Evaluation	2019	Johnson J, DelGuidice B, Bangari DS, Peterson E, Ulinski G, Ryan S, Thurberg BL, Callis G	CRC Press	Anatomical basis for the location of principal anatomical structures in mouse
X-Ray Annotation Mouse Atlas	2021	Ruberte J, Carretero A, Cater H, Gracia G, Lally C	Doctor Herriot SL	Atlas. 152 X-rays and 590 anatomical references. Uses the Nomina Anatomica Veterinaria
Essentials of Laboratory Animal Science: Principles and Practices	2021	Nagaran P, Ramachandra G, Srinivasan R	Springer	One chapter devoted to mouse anatomy and physiology
Pathology of Genetically Engineered and other Mutant Mice	2022	Sundberg JP, Vogel P, Ward JM	Wiley Blackwell	Several chapters with anatomical introductions

Advances in imaging and high resolution phenotyping in recent years have generated a need for a granular and complete anatomical terminology for the mouse which is cognate to that available for humans. To answer this need we conclude this commentary by discussing the development of a standard anatomical nomenclature for the mouse following the principles of the TA terminology. Because the mouse and human should have similar complexity, this mouse Terminologia Anatomica (mTA) should have a number of normalised anatomical concepts similar to that of the TA, and would greatly assist in the further development of the adult mouse anatomy ontology and related resources.

## Mice are a small animal model but anatomically similar to humans

Recognition of the anatomical and physiological similarities and commonalities between different species can be traced in the literature to Aristotle who probably reflected established opinions when he wrote in the 4th C BCE:

“*Ought we, for instance, to begin by discussing each separate species-man, lion, ox, and the like—taking each kind in hand independently of the rest, or ought we rather to deal first with the attributes which they have in common in virtue of some common element of their nature, and proceed from this as a basis for the consideration of them separately*” (Περὶ ζῴων μορίων; On the parts of animals. Aristotle. Trans. William Ogle, 1882).

The common anatomy, physiology and ontogeny of species closely related to humans has made mammals the obvious model organisms for increasing our understanding of human biology and disease since the Greco-Roman period, and arguably earlier. Galen (129–216 CE) compared the surgical anatomy of wounded gladiators from the arena of Pergamum and of his patients in Rome, with that of primates, goats and pigs. Using the opportunities presented by dead and living individuals of all species, including occasionally living humans, he attempted to establish the function of anatomical structures and their involvement in disease, and initiated a long tradition of developing surgical practice using animal models (Matter 2013).

Since Galen and his Greek precedents, animal models have historically played a critical role in the exploration and characterization of disease and in the development of novel therapeutic agents and treatments, now being called the One medicine, One pathology, One health concept (Sundberg and Schofield 2009). The mouse has now become the most widely used, and arguably the most important, mammalian model for studying human disease. Mice are biologically very similar to humans and manifest many of the same or closely-related diseases (McGonigle and Ruggeri 2014). Most significantly, work since the beginning of the twentieth century, on generating genetically inbred strains of mice, has established the mouse as the most powerful organism for discovery of the genetic basis of disease. Mapping of spontaneous genetic variation between mouse strains, the experimental modification of the genome, and the ability to manipulate processes of development has greatly contributed to our understanding of the molecular mechanisms of human disease, and allowed the creation of models of human diseases that can be used for the development of therapeutics and further our understanding of pathobiology (Vandamme 2014). Furthermore, mice are a cost-effective research tool. They have a short inter-generational lifespan, relatively short longevity, and they are easy to house and transport at scale.

Mice are biologically very comparable to humans, have ~ 15,000 genes with human orthologues, and are therefore susceptible to the majority of mono- and polygenic inherited disorders affecting the human population. Mice can be genetically manipulated to mimic the causative or contributing genetic mutation or mutations underlying many human disease conditions (Brown 2021). Furthermore, mice are a cost-efficient research tool (Leader and Padgett 1980) with an accelerated lifespan (one mouse year equaling ~ 30 human years), small footprint animal holding, and are relatively easy to handle and transport; particularly as frozen germ plasm.

**Fig. 1:**
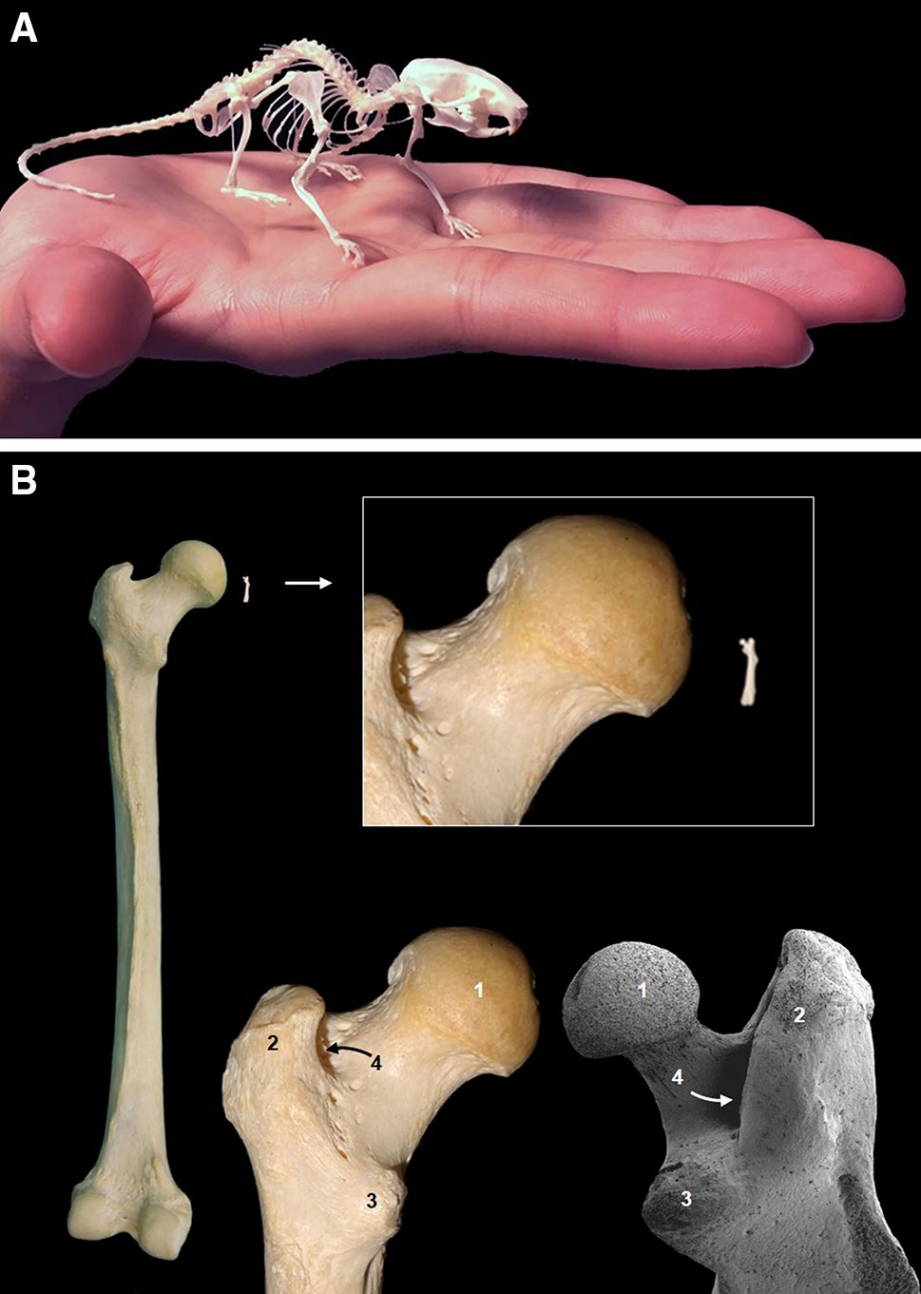
HYPERLINK "sps:id::fig1||locator::gr1||mediaobject::0" **A** Skeleton of a mouse relative to the human hand. **B** Comparison of the human and mouse proximal epiphyses of the femur. Similarity of the anatomical structures of the head of the femur (1), great trochanter (2), lesser trochanter (3), and trochanteric fossa (4) using scanning microscopy and image size correction for the mouse. Mouse images modified from Ruberte et al. (2016) with permission

Although in comparison with humans, mice are small (Fig. 1A), they display a relatively similar anatomy. As an example of this, Fig. 1B shows a comparison between the human and mouse femur; the latter enlarged using scanning electron microscopy. The same gross anatomical details: head of the femur, the lesser and great trochanter, as well as, the trochanteric fossa, can be observed both in the proximal epiphysis of human and mouse femur. These anatomical similarities not only represent a morphological homology between these two mammalian species, but they also reflect similarities in the biomechanics and function of the bone. The comparatively larger size of the head of the femur in humans reflects the different body positions of both species (Fig. 1B). Humans are bipedal and the entire body weight is transmitted through the femoral heads to the ground. By contrast, in the mouse, a quadrupedal species, the body weight is transmitted to the ground through all four limbs.

**Fig. 2:**
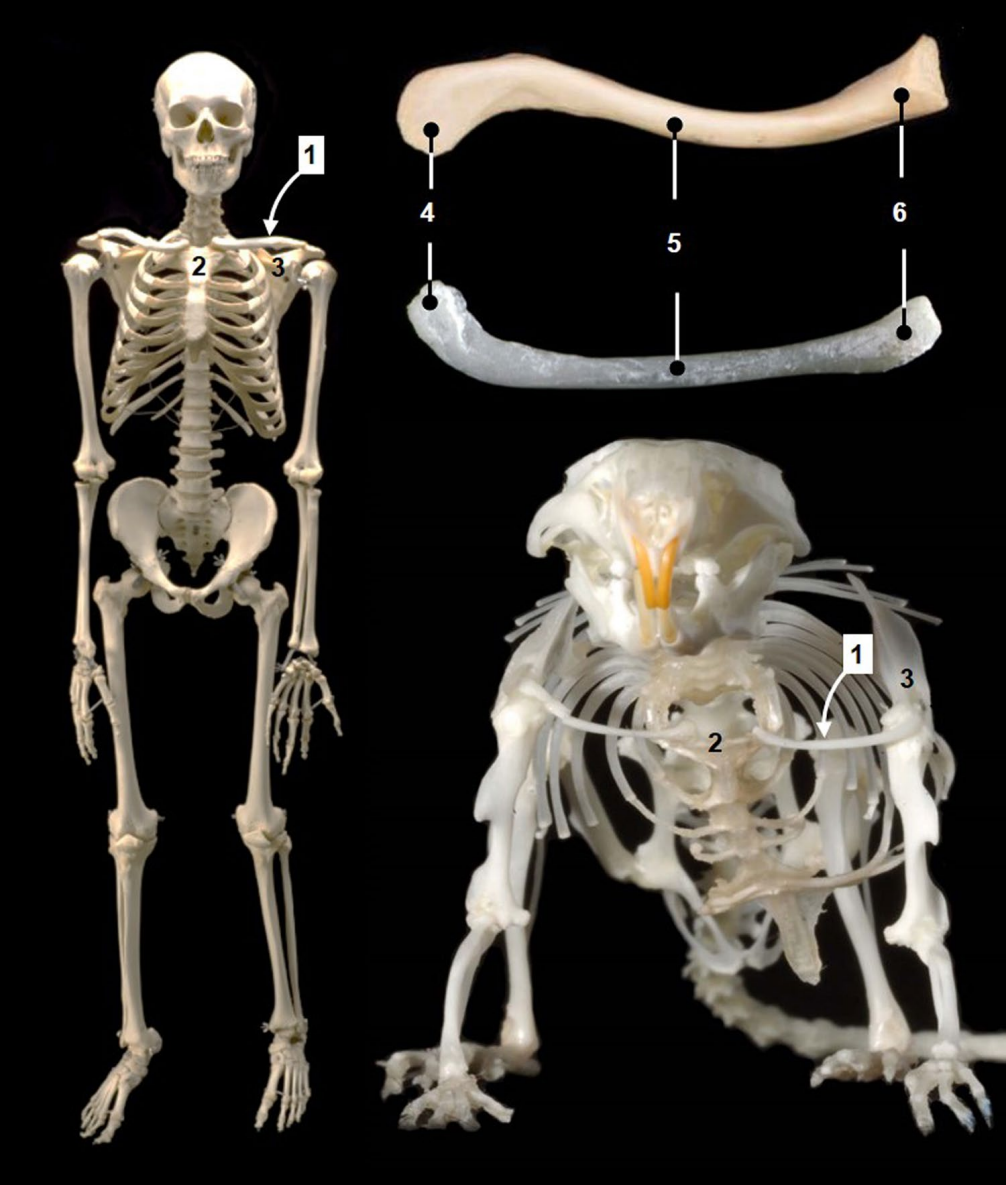
The quadrupedal mouse has unexpected anatomical similarities to the bipedal human, such as the presence of the clavicle (1). The clavicle in both attaches the sternum (2) and the acromion of the scapula (3). Acromial end (4), body of clavicle (5), esternal end (6). Mouse images modified from Ruberte et al. (2016) with permission

Sometimes, anatomical similarities between humans and mice are unexpected (Fig. 2). In bipedal primates and humans, the clavicle, a bone belonging to the pectoral girdle, is well-developed because there is an adaptation to tasks that depend on using hands distant from the central trunk axis, such as climbing and reaching distant objects (Rockwood et al. 2009). By contrast, in many quadrupedal mammals, such as ungulates, carnivores and several rodents, the clavicle has disappeared or has been dramatically reduced (De Souza et al. 2020). This reduction or loss is common to mammals that use the thoracic limb for cursorial locomotion (Rockwood et al. 2009). However, the quadrupedal mouse has a well-developed clavicle that, as happens in man, joins the sternum and the acromion of the scapula (Fig. 2). A possible reason for this unexpected anatomical similarity may be the consequence of the thoracic limb freedom that mice have. Mice are often seen standing on their hind limbs trying to climb their cage walls and this rearing action is associated with exploration and manipulation of the environment. Recordings using high-speed close-up video of mice eating seeds (Barrett et al. 2020) show well developed manual dexterity for manipulating small objects.

**Fig. 3:**
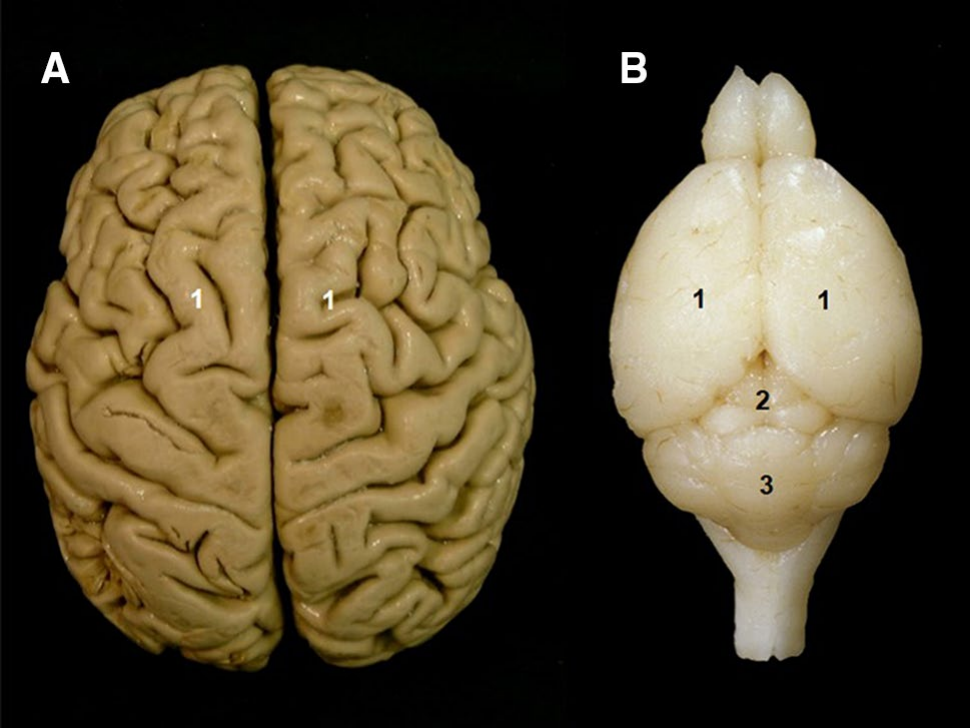
Mouse and human have anatomical differences in the brain. **A** The human cerebral cortex is gyrencephalic with characteristic gyri (ridges) and sulci (depressions or furrows). **B** The mouse cerebral cortex is lissencephalic with a smooth surface and the telencephalon (1) does not cover the mesencephalon (2) and cerebellum (3). Mouse image modified from Ruberte et al. (2016) with permission

**Fig. 4:**
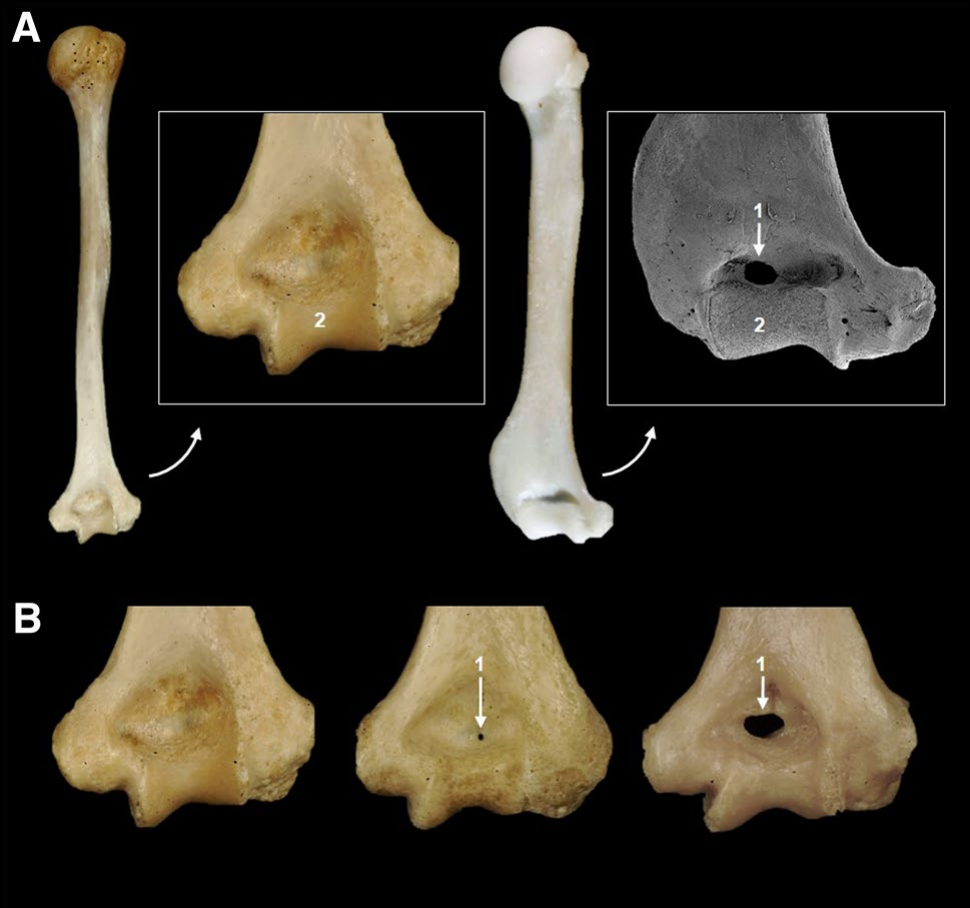
**A** A previously purported anatomical difference between mouse and human humeri is the presence of a supratrochlear foramen (1) in the humerus of the mouse but not in the human humerus. **B** Variable presence of the supratrochlear foramen in humans. Trochlea of humerus (2). Mouse image modified from Ruberte et al. (2016) with permission

Despite these similarities, mice and humans do present clear anatomical differences. These manifest at the macroscopic, microscopic and system levels. For example, the sexual dimorphism of the mouse kidney is well known (Butterfield 1972). One of the most notable macroscopic differences lies in the mouse brain, which in human anatomical terms is lissencephalic, since its cerebral cortex does not have folds (gyri), and its telencephalon does not completely cover the mesencephalon and the cerebellum, as happens in humans (Fig. 3). Despite these overt anatomic differences, the mouse has nevertheless contributed enormously to our understanding of human lissencephaly and its underlying genetics, reflecting fundamental similarities in the development of the CNS between the species (Del-Valle-Anton and Borrell 2022; Mota and Herculano-Houzel 2015). There are other very well-established anatomical differences, such as the presence of a characteristic supratrochlear foramen in the mouse humerus (Fig. 4A), however, on close consideration this turns out not to be a real difference to humans. Proximal to the trochlea of the human humerus there is a thin region of bone that is sometimes perforated forming a supratrochlear foramen like the mouse (Fig. 4B). The prevalence of this foramen varies from 6.5% in Europeans (Mays 2008) to 26.7% of people from India (Shivaleela et al. 2016). Functionally, a supratrochlear foramen in human allows hyperextension of the elbow (de Wilde et al. 2004). Women who are more flexible than men and people that generally practice disciplines that need improved flexibility, such as yoga, have higher prevalence of a supratrochlear foramen (Akabori 1934; Shivaleela et al. 2016). Similarly, it is reasonable to suppose that the supratrochlear foramen in mice provides the elbow with the capacity to hyperextend. It is just as likely that careful examination of this structure in the new, highly genetically variable, collaborative cross strains (Collaborative Cross 2012) and Diversity Outbred mice (Churchill et al. 2012) will identify a wide variety features that do or do not resemble human anatomy.

As discussed below, the rate of discovery of new anatomical structures and entities in humans is now quite slow, but several recent discoveries were paradoxically made first in the mouse. For example, the recent discovery of functional lymphatic vessels lining the dural sinuses originated in detailed functional and molecular studies in the mouse CNS (Aspelund et al. 2015; Eide et al. 2018; Louveau et al. 2017, 2015). Understanding the functions and relationships of the glymphatic system and meningeal lymphatics (Eide et al. 2018). Similarly, with the discovery of the network of transcortical capillaries in long bones that now helps to explain previous puzzles in the migration of neutrophils and the closed circulation system of bone (Grüneboom et al. 2019). It is noteworthy that in both of these cases the development of imaging techniques and the unique ability to use molecular markers, tissue preparations and experimental procedures in the mouse facilitated these discoveries. Are we moving into an era of discovery of new human anatomical features from the mouse or as a result of better analytical tools?

## Mice and humans: a similar anatomical complexity but different level of knowledge and resources

Knowledge about human anatomy has been collected throughout history. Since Galen's anatomical descriptions, and even before, up to the present, the level of detail found in human anatomical books, and other resources has continuously increased. This is especially true for anatomic variations. The classic, Bergman’s Comprehensive Encyclopedia of Human Anatomic Variation (Tubs et al. 2016), gives some an idea of the current level of knowledge; 1456 pages and more than 12,500 references.

An anatomic variation can be defined as difference in morphology that is outside of the canonical. It is important to remember, however, that the canonical is often an accepted abstract standard, as the dominant morph may well be different for genetically different populations. For example, the supratrochlear foramen in human humerus was first described two centuries ago (Meckel 1825). By contrast, the first comprehensive study of mouse anatomy was published by Margaret J. Cook in 1965. Cook worked at the MRC Laboratory Animal Centre in Carshalton (UK) and carried out intracardiac latex injections describing for the first time the mouse arterial and venous system. In Cook’s atlas foreword (Cook 1965), W. Lane-Petter stated that at that time no complete study of the anatomy of the mouse had so far been made and that gap in the knowledge was even more surprising as the mouse was at that time the most commonly used vertebrate in the laboratory.

Subsequent to the publication of Cook’s atlas, several comprehensive textbooks and other resources in mouse anatomy and histology, singly or as part of larger compendia, have been published. Table 1 lists the title, year of publication, authors, publisher, and characteristics of the extant resource. However, despite the steady release of textbook or on-line resources since Cook’s seminal publication, the depth of detailed description and understanding of variation in mouse anatomy is far from comparable to human anatomy thereby presenting a challenge for using the mouse as a comparable and informative model of human biology and disease.

## Of originals and copies: the case of bulbourethral glands

Anatomy is perceived as an objective scientific discipline, because it involves the observation and description of definable material entities. However, dissection, the most preeminent technique to physically dissociate and isolate individual structures for examination is, like other analytical scientific methods, susceptible to artifact. The discovery of bulbourethral glands in the mouse and the historical way to understand its morphology and topography is a good example of how dissection can be misleading, and that anatomy is not always as obvious as the “nose on your face”.

**Fig. 5:**
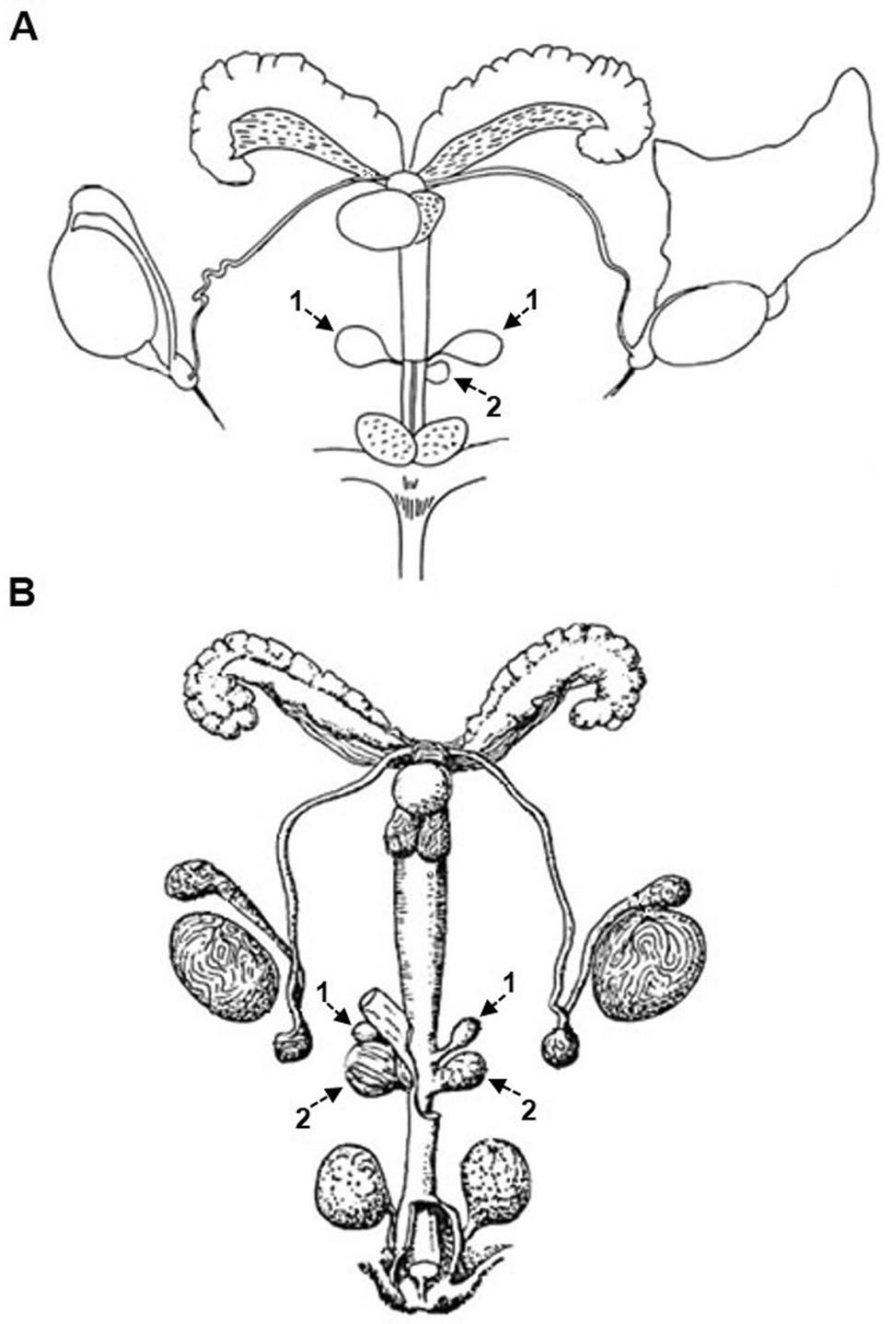
Comparison between Cook’s and Fekete’s representations of mouse bulbourethral glands. **A** Cook described paired glands (1) with only one urethral recess (2). **B** Fekete described two paired glands (1) and two paired urethral diverticula (2) (adapted from Cook 1965 and Snell 1941)

The bulbourethral glands are part of the male accessory genital glands, which are located along the pelvic portion of the urethra producing secretions for the nutrition, transportation and protection of spermatozoa. Not all mammals have bulbourethral glands. The dog lacks bulbourethral glands, which might explain why they were missed in Cook's book. In her atlas, six drawings were devoted to the male genital system, however, bulbourethral glands were not noted. In fact, it would be necessary to wait until 1983, when Cook, in a book about the use of mouse in biomedical research (Cook 1983) showed and drew the location of two paired bulbourethral glands emptying into the urethra and associated with a urethral diverticulum (Fig. 5A). From that time, several classic and modern mouse books used the same figure to locate and describe these glands (Maronpot et al. 1999; Treuting et al. 2018). However, this anatomical description for the mouse bulbourethral glands is not the only one that appears in textbooks. Several years before the publication of Cook´s atlas, The Jackson Laboratory published its celebrated book: Biology of Laboratory Mouse (Snell 1941) in which, E. Fekete, who at that time was responsible of the histology laboratory, carried out a different anatomical description for the mouse bulbourethral glands. Fekete’s representation showed the bulbourethral glands related with two urethral diverticula, each of them covered by the bulbospongiosus muscle. As happened with Cook's description, Fekete's anatomical interpretation was later followed by Popesko et al. (1992) in its beautiful anatomy atlas of laboratory animals, and subsequently Popesko’s drawings were copied by V. Komàrek in the first and second editions of the Laboratory Mouse book (Hedrich 2012; Hedrich and Bullock 2004). Furthermore in 2011, G.M. Constantinescu, Professor of Veterinary Anatomy and Medical Illustrator at the University of Missouri-Columbia, carrying out new original anatomical preparations for its book “Comparative Anatomy of the Mouse and the Rat” (Constantinescu 2011) described mouse bulbourethral glands as did E. Fekete in 1941.

**Fig. 6:**
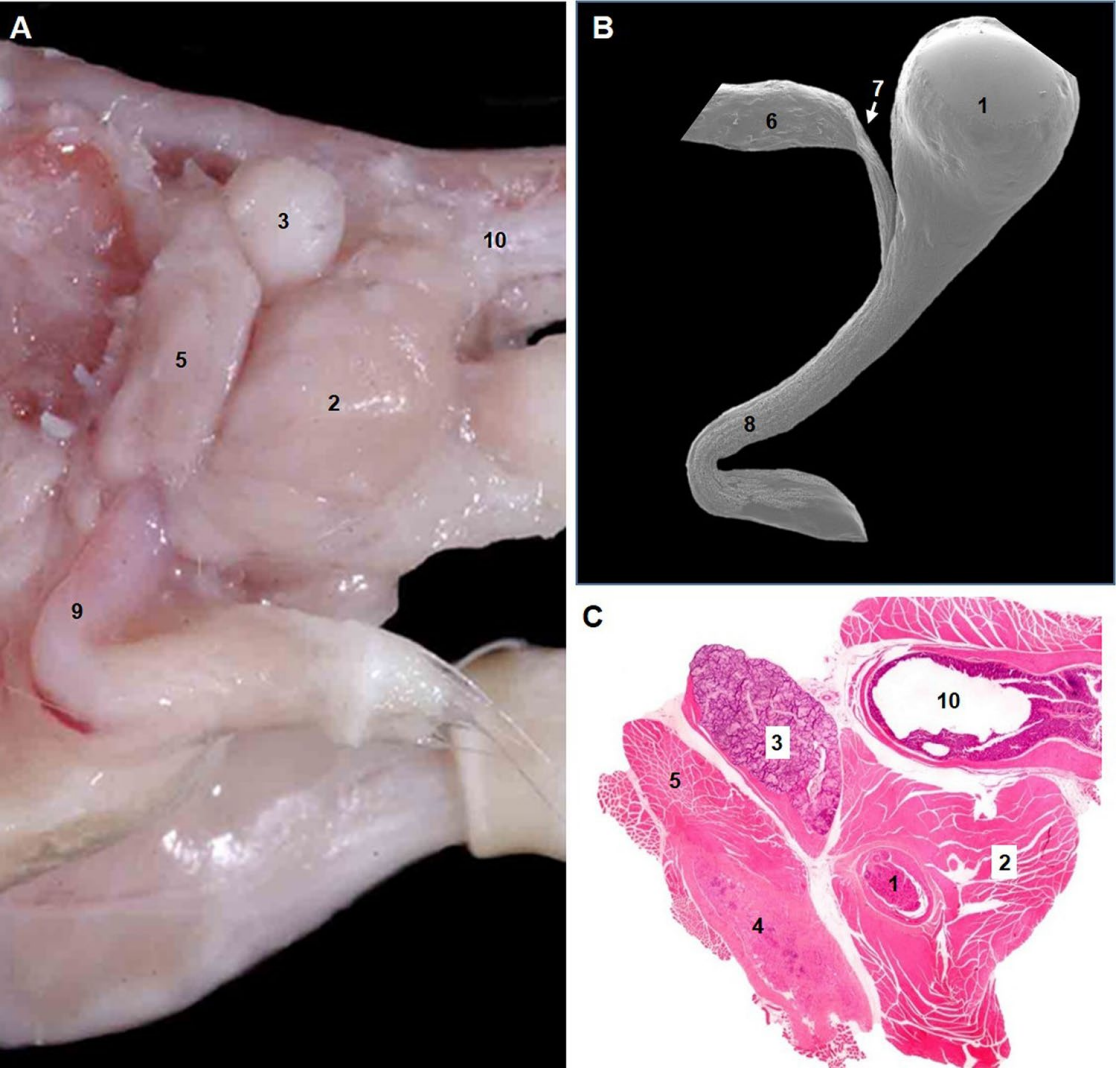
Analysis of the urethra in C57BL/6J male mice identified only one urethral recess within the bulbospongiosus muscle. **A** Gross dissection of the pelvis (lateral aspect). **B** Scanning microscopy of corrosion cast of the urethra (lateral aspect). Hematoxylin and eosin stained histological section of the root of the penis. Urethral recess (1); bulbospongiosus muscle (2); bulbourethral gland (3); corpus cavernosum (4); ischiocavernosus muscle (5); urethra membranosa (6); urethral isthmus (7), penile urethra (8); penis (9); rectum (10). Mouse images modified from Ruberte et al. (2016) with permission

The last macroscopic organ discovered in humans was the cisterna chyli, that was first described in 1651 by the French physician Jean Pecquet, as a lymph reservoir at the origin of the thoracic duct (Natale et al. 2017). In other words, for almost four hundred years we have had a very complete description of human macroscopic anatomy. By contrast, in the mouse we have severe discrepancies in anatomical descriptions for important organs, such as the bulbourethral gland (Fig. 5). One reason for these discordances could be that the classic technique used to collect the accessory genital glands in male mouse, a ventral approach after opening the abdominal cavity, is not optimal to identify and dissect the bulbourethral glands, which are located laterally at the base of the tail between the crus and the bulb of the penis (Fig. 6A). This difficulty was brought to light in several mouse histology books, such as the one published in 2014 by C.L. Scudamore (Scudamore 2014), which comments that in standard necropsy sampling protocols, the bulbourethral glands are usually missing. The difficulty of dissecting the bulbourethral glands is also obvious, when in a recent published guide for the location and orientation of mouse tissues for optimal histological evaluation (Johnson et al. 2019), no specific instruction is provided to dissect and sample the bulbourethral glands. However, the abnormal swelling of these glands, that presents with male mice seeming to have “4 testicles”, enables easy identification of these structures such that they can be dissected and studied (Kiupel et al. 2000).

**Fig. 7:**
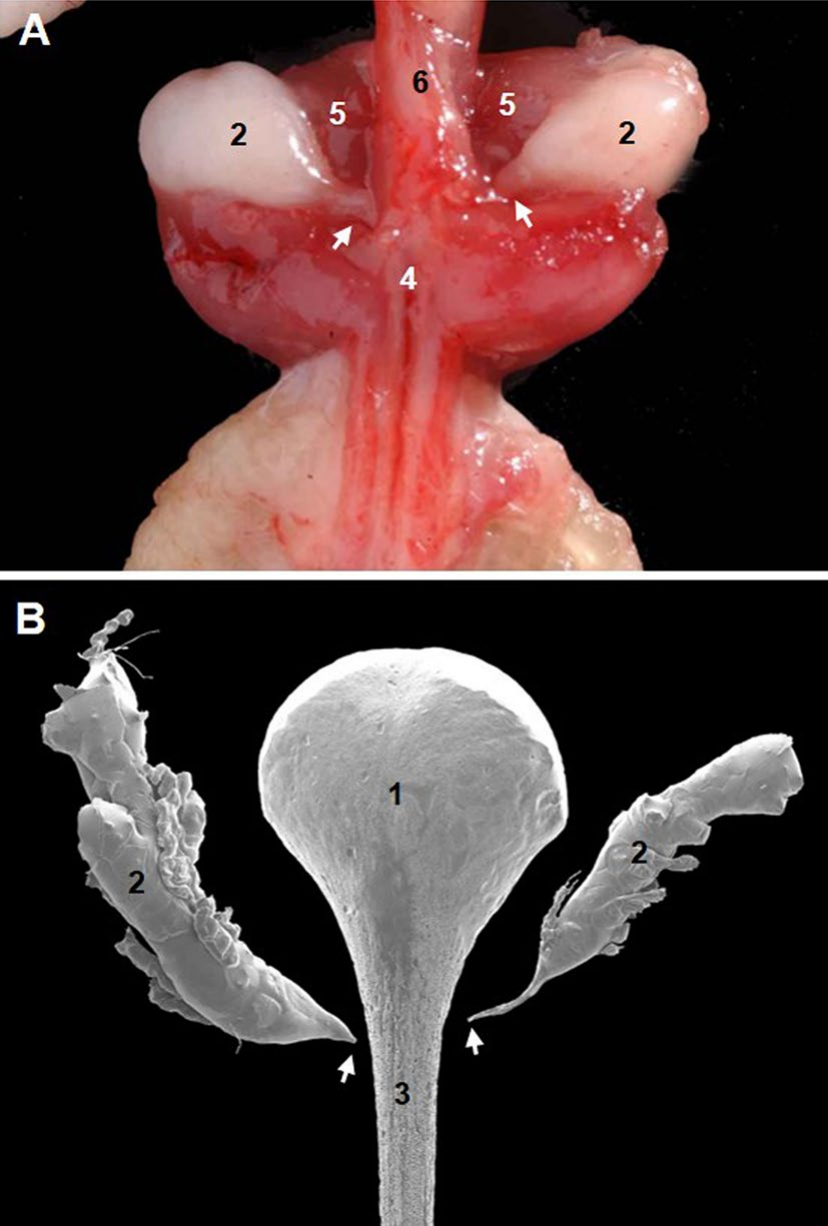
**A** Gross dissection of the root of the penis (caudal aspect). **B** Scanning microscopy of corrosion cast of the urethra (caudal aspect). Urethral recess (1); bulbourethral gland (2); penile urethra (3); bulbospongiosus muscle (4); ischiocavernosus muscles (5); urethra membranosa (6); bulbourethral gland ducts to the penile urethra (arrows). Mouse image modified from Ruberte et al. (2016) with permission

To understand the anatomical relations of bulbourethral glands in the mouse and to clarify which of the two morphological representations is more accurate, injection of a polymerizable resin (Mercox^®^) in the urethra of C57/BL6J male mice was done via the urinary bladder. The four casts obtained were immersed in 60 °C soapy water for 24 h, corroded in 3% KOH and washed in distilled water. Casts were mounted on stubs, sputtered with gold and observed in a Hitachi S-570 scanning electron microscope at an accelerating voltage of 10–15 kV. Analysis of the urethral casts (Figs. 6B and 7B) and correlative dissections and histological sections (Figs. 6A, C and 7A) showed that the pelvic part of the urethra in mice, as happens in humans, presented a large membranous portion. There is a narrowing of the urethra when it turns around the ischial arch, the urethral isthmus, which is well documented in domestic mammals (Fig. 6B). At the penile urethra, inside the bulb of the penis, only a single urethral recess was noticed (Figs. 6B and 7B). The histological sections showed that this diverticulum is covered by the bulbospongiosus muscle and surrounded by a narrow band of erectile tissue corresponding to the corpus spongiosum (Fig. 6C). The two corpora cavernosa, that complete the penile erectile system, are inside the crura penis covered by the ischiocavernosus muscles (Fig. 6C). The ducts of the two bulbourethral glands join the penile urethra distal to the urethral recess (Fig. 7). The urethral recess of the mouse has a homonymous dilation of the urethra in male humans. Our study suggests that the anatomical description of Cook (1983), although incomplete, is more accurate (Fig. 5). Only one diverticulum is present in the mouse urethra and not two as Fekete (1941) and her followers point out.

## Mouse vs human anatomical terminology and nomenclature

Terminology is understood as a set of terms used in a specific scientific field, whereas nomenclature is a normalized system of exactly defined terms arranged according to certain classification principles. A nomenclature is approved and refined by a scientific commission and is widely accepted by the professional community (Kachlik et al. 2008).

The origins of anatomical terminology date back to the ancient period, more than 2500 years ago, however, the first effort to compile a unified anatomical terminology, the Basle Nomina Anatomica, was made in 1885 (His 1885). At that time, the number of human anatomical terms reached 50,000, many of them synonyms. Structures and organs were given eponyms from the person first credited with the first description, and in many cases the same organ was associated with the names of different anatomists in different countries (O’Rahilly 1989). This first Nomina Anatomica was followed by seven revisions. The last revision was published with the title of Terminologia Anatomica (TA) and consists of a list of terms in Latin including their corresponding translation in English (F.I.P.A.T 2019). The TA is not applicable to domestic animals because the terms of direction were based on the erect position of the human body and not in the quadrupedal position of animals, like the mouse. Therefore, a Nomina Anatomica Veterinaria (NAV) was established and today it is in its sixth revised edition (I.C.V.G.A.N. 2017).

There are 7635 terms in the TA and 6500 in the NAV, that are equivalent and have the same principles: (1) each structure should be designated by a single term; (2) each term in the official list is in Latin, but with liberty to translate; (3) each term is, so far as possible, short and simple; (4) the terms are primarily memory signs, but do have some informative or descriptive value, and (5) terms derived from proper names (eponyms) should not be used.

Largely driven by the advent of high throughput gene expression analysis it became important to capture knowledge about anatomy that might be used computationally across very large datasets. This need was further reinforced by the development of large phenotype databases for the mouse which needed a formally computable structure in order to search it. Very rapidly it became apparent that these needs would be answered by the development of formal ontologies to describe anatomy, at all levels, and phenotype—the latter necessarily needing to incorporate anatomy into its concepts, and pathology where the location of a lesion needed to be captured.

For these needs to be met, the “anatome”, the complete set of anatomical structures associated with an organism, needed to be organized in a computer-comprehensible way in order to support inference, reasoning, and estimates of semantic similarity. Lists of anatomical terms alone, like TA and the NAV, are inadequate for this formalization because anatomical nomenclatures cannot easily put in the format appropriate for the structure of relational databases (Bard 2005).

The solution adopted was to organize the anatomy of each organism in a hierarchy of anatomical structures and linking relationships (e.g., the bulbourethral gland *is_a* male reproductive gland and is *part_of* male urethra). The adoption of a formal ontology to describe the domain of anatomy has very important advantages over other approaches and allows the use for formal semantic methods to make inference, subsume related concepts under higher concepts (parents) and to use automated reasoning. The advent of the Ontology Web Language (OWL) for most bio-ontologies allows for much more precise and expressive modelling of the concept domain, with restrictions, properties, instances and complex relationships that allow the capture of a great deal of knowledge. While an ontology is not intrinsically a graph, it may be expressed as such, especially as humans are familiar with navigating hierarchies of concepts—a knowledge structure that goes back to the *Etymologiae* of Isidore of Seville in the sixth century CE (Barney et al. 2006). A formal ontology allows for modelling of the knowledge within it and it is interesting that here several anatomical ontologies, notable the Foundational Model of Anatomy (FMA) ontology (Rosse and Mejino 2008), the time-dependent embryological anatomy ontology of the mouse (EMAPA) (Hayamizu et al. 2013) and the Mouse Anatomy (MA) ontology, differ. As we will see below these differences in modelling decisions make it complicated to map and align these ontologies, specifically the FMA, which is important for integrating human and mouse data. The development of the Uberon metazoan ontology (Mungall et al. 2012) (https://obofoundry.org/ontology/uberon) was designed to bridge the anatomical ‘silos” particularly between vertebrates.

The first attempt to develop a mouse anatomy ontology was an internet-accessible database of names and synonyms of the tissues in the first 22 Theiler stages of development (Bard et al. 1998). A few years later, an adult Mouse Anatomy (MA) ontology was developed in The Jackson Laboratory (Hayamizu et al. 2005a). MA ontology contains 3300 unique terms to provide standardized nomenclature for anatomical structures in the mouse. The MA ontology can be accessed at the Mouse Genome Informatics website:

(MGI, https://www.informatics.jax.org/vocab/gxd/ma_ontology).

MA is a polyhierarchy with several different axes of organization. These include anatomic region organ system, organ, substance, and tissue. The grouping of individual organs and structures by function or physiology, for example the duodenal glands (formally called Brunner’s glands) *is_a* exocrine gland and is *part_of* the small intestine.

The MA Browser enables one to navigate through the ontology in two ways. Progressively, scrolling through the various hierarchies and in the Term tab obtaining information about individual terms, including ID and relationship to other terms. Alternatively, an anatomy search could be done introducing any text sequence in the query field. Then, all terms in the MA vocabulary, including any synonyms, contained in text will be displayed (Hayamizu et al. 2005a, 2015). The MA ontology can also be downloaded from the Open Biomedical Ontologies website (OBO; https://sourceforge.net/projects/obo). The MA ontology is currently used to annotate: (1) developmental expression data in the Gene Expression Database (GXD; http://www.informatics.jax.org/expression.html) (Smith et al. 2015); (2) phenotype data obtained in the International Mouse Phenotyping Consortium (IMPC; https://www.mousephenotype.org) (Elmore et al. 2018); and (3) pathology data in Pathbase (http://www.pathbase.net) (Schofield et al. 2004). Post-natal mouse anatomy terms are also available as Theiler stages 27 and 28 in the EMAPA ontology where developmental anatomy is available in the same hierarchy as adult/postnatal (Hayamizu et al. 2013).

No scientific terminology can be considered complete and permanent as long as research in the field continues, and the MA ontology like others in the OBO Foundry continues to be developed by its curators in response to new applications and the discovery of new knowledge. MA ontology developers have continued to refine terminology, adding synonyms, and revising anatomical concepts. However, due to the increasing number of publications and books dealing with mouse anatomy published in recent years (Table 1) there is a need to continuously develop the ontology and to align mouse and human anatomy.

We propose the development of a mouse version of the Terminologica Anatomica with high resolution detail, structured and consistent with the principles established in the TA and the NAV, the mTA. One example of how such a terminology might deal with anatomical complexity and existing standards is for the carpal bones.

**Fig. 8:**
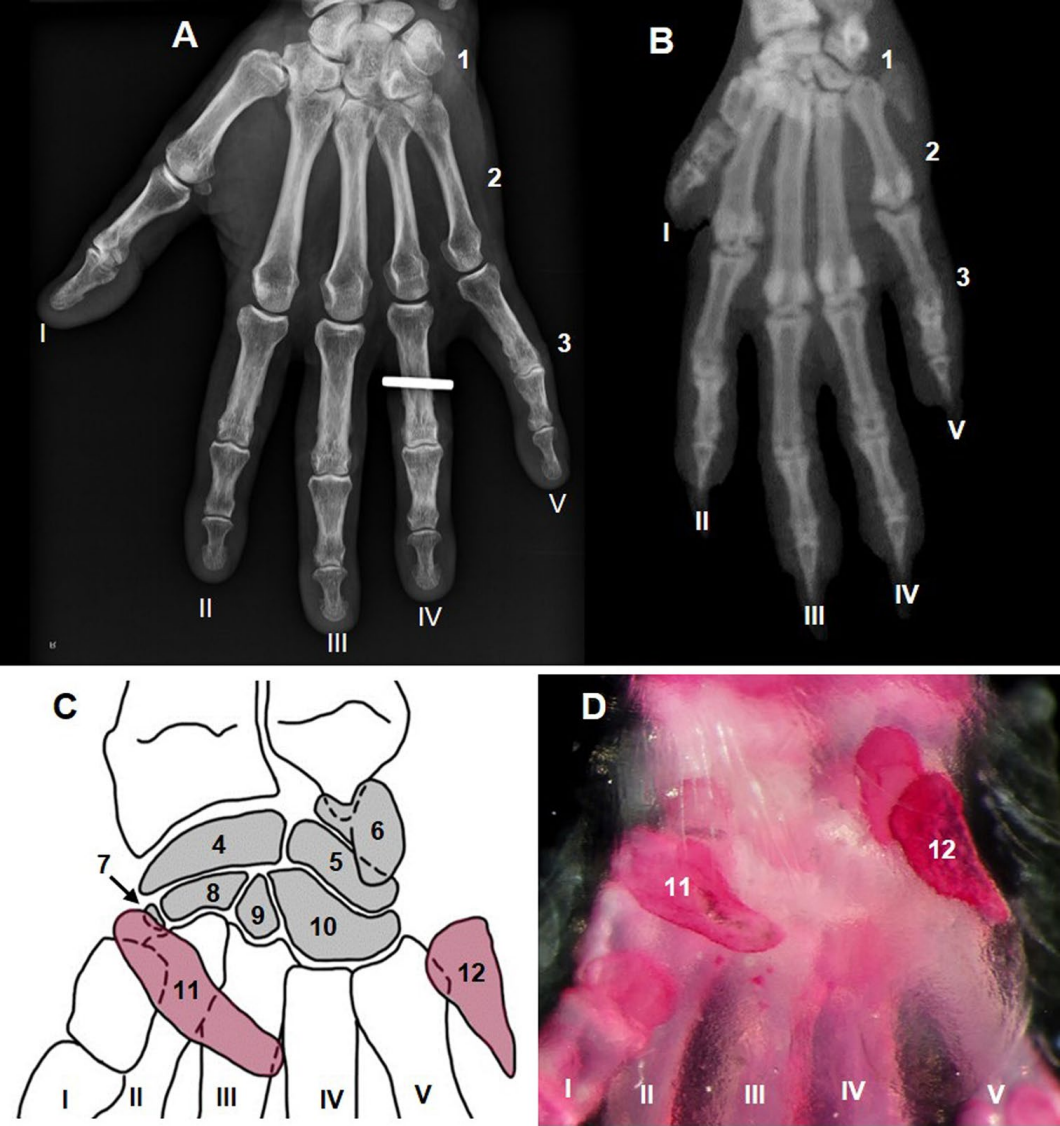
Dorsal ventral radiographs of human hand (**A**) and mouse forepaw (**B**). Diagram showing the topography and organization of mouse carpal bones (**C**). Alizarin stained mouse carpus (palmar aspect) (**D**). Carpal bones (1); metacarpal bones (2); phalanges (3); scapholunate (4); triquetral (5); pisiform (6); trapezium (7); trapezoid (8); capitate (9); hamate (10); falciform carpal bone (11), which is a sesamoid bone embedded in the flexor retinaculum and not a true carpal bone; ulnar sesamoid bone (12). Roman numerals indicate the medial to lateral order of digits

Carpal bones in mice are arranged in two rows (Fig. 8). There are several aspects that could be considered regarding this hierarchy and terminology. First, the falciform carpal bone is not a true carpal bone, in fact is a sesamoid bone embedded in the flexor retinaculum (Wirtschafter and Tsujimura 1961), therefore, it should not be considered to be a carpal bone. Furthermore, carpal bones in mouse present many anatomic variations (Ruberte et al. 2021). In 15% of 28 C57BL/6J carpals analyzed a supernumerary bone, the central carpal bone, was observed between the proximal and distal row. Additionally, this bone was fused to the scapholunate (in 45% of cases) and to the trapezoid (40%), thus the central carpal bone should be considered, as happens in the TA, as an actual carpal bone in mouse. It is a common practice to number the carpal bones from medial to lateral, first in the proximal row and then in the distal row. This procedure was first adopted by Vesalius (Vesalius 1543) and after that all anatomy resources have followed this rule, including TA and NAV.

As a corollary to the previous comments, it seems clear that it would be useful to integrate or map terms in an mTA to mouse anatomical terms in the MA and EMAPA ontologies, using community effort. Table 2 shows the comparison of the current terminologies with the proposed mTA. The great advantage of the Open Biomedical Ontology principles is that they encourage community input to the development and continuous refinement of ontologies by the user community. Because the mouse, domestic animals, and humans have a similar anatomical complexity, mTA probably should have a number of terms similar to those of the TA and NAV, around 6000, considerably increasing granularity, although attention needs to be paid to structuring classes in both terminologies when considering these absolute numbers. More importantly formal mapping or axiomatization of MA to FMA could also be considered along with cross references to classes in the human Terminologica Anatomica. We are confident that this development would greatly support morphological phenotyping of mouse models as modern imaging methods increasingly need a higher degree of anatomical granularity for annotation than is currently available.

**Table 2:** Comparison between current anatomical terminologies/ontologies an the proposed mTA

Terminology/Ontology	Scope and Focus	Structure and organization	Annotation and detail	Applications	No of classes	Ontology depth	Axes of classification	Formal ontology (OWL)	Knowledge base/terminology	Asserted references/imported classes	Authority
UBERON	Multi-species anatomy ontology that aims to provide a common framework for representing anatomical structures, both adult and developmental, across diverse organisms, including humans, mice, and other model organisms	Built on the principles of the Open Biomedical Ontologies (OBO) Foundry, using a hierarchical structure of classes and relationships to represent anatomical entities, cells tissues and features across different species. Includes many relationship types such as 'innervated_by", "part_of" etc	Aims to provide a broad coverage of anatomical entities across species but may not have extensive detail at the level of individual species or developmental stages	Widely used in bioinformatics and comparative anatomy studies to integrate and analyze anatomical data across species and to support cross-species research	25,930	34	Complex hierarchy containing mixed biological concepts and semantic types; not all anatomical Spatial, taxonomic, developmental and systematic relations	Yes	No	Refers to or imports more than 30 anatomy, species specific, molecular and functional ontologies, e.g. GO, ChEBI, MA, FMA etc. Specifies presence of classes in taxon	Uberon curation team
MA	Specifically focuses on the anatomical structures of the adult mouse, providing detailed information about the mouse anatomy at the macroscopic and microscopic levels	Uses a hierarchical structure to organize anatomical structures within the adult mouse, including regions, organs, and tissues. It provides specific information relevant to the mouse anatomy. "is_a" and "part_of"	Provides detailed annotations specific to the anatomy of the adult mouse, including information on organ systems, structures, and their spatial relationships	Primarily used in mouse research, such as studies involving mouse models of human diseases or investigations into the mouse anatomy for experimental design and interpretation	3257	9	Anatomically systematic. Region, organ system, body fluid, substance, organ	Yes	No		MA local curation team, The Jackson Laboratory
FMA	Focus on human anatomy, providing a comprehensive representation of human anatomical structures and their relationships	Employs a complex structure based on a foundational model, utilizing a mix of hierarchical relationships, partonomic relationships, and other axioms to represent human anatomical entities and their attributes	Provide comprehensive and detailed information about human anatomical structures, including spatial relationships, hierarchical organization, and functional attributes	Extensively used in various human clinical fields, including medical education, clinical informatics, imaging and anatomical research, to provide a detailed and standardized representation of human anatomy	104,721	23	Anatomically systematic. Physical and non physical anatomical entities, including relations and transformation entities	Yes	No		FMA local curation team
NAV	NAV aims to establish a standardized nomenclature for anatomical structures in veterinary medicine covering the main veterinary species. Covers a wide range of animal species, including cat, dog, pig, cattle, sheep, goat, horse, and rabbit	NAV is organized systematically, providing hierarchical relationships between anatomical structures. Terms are classified based on their position, function, and relationship with other structures. This hierarchical organization allows for clear identification and classification of anatomical entities	NAV includes species-specific terms to address anatomical variations and differences among different animal species. It recognizes that animals may have unique anatomical features, and the terminology provides species-specific terms to accurately describe those structures	It provides a common language and set of terms for accurately and consistently describing anatomical structures in animals. Used by veterinarians, researchers, and anatomists mainly in the clinical domain. Global reach and available in Latin and English	> 6500	N/A	Hierarchical structure. General divisions on major anatomical systems or regions of the body, specifc regions or structures within each general system. Hierarchical relationships captured and species specific terminology	No	Yes		Maintained and updated by the International Committee on Veterinary Gross Anatomical Nomenclature (ICVGAN)
mTA	mTA aims to establish a standardized nomenclature for confirmed anatomical structures in laboratory mice (*Mus musculus*)	mTa will be organised systematically in several sections: general terms, regions of the body, osteology, arthrology, myology, digestive apparatus, respiratory apparatus, thoracic cavity, urogenital apparatus, urinary organs, male and female genital organs, perineum, peritoneum, endocrine glands, angiology, nervous system, and common integument. Anatomical terms are classified based on their position, shape, function and relationship with other structures	mTA will provide definitions of mouse anatomical terms and its relation to human anatomical concepts and structures	It will provide a set of confirmed anatomical structures for accurately and consistently describing mouse morphology. Definitions of anatomical terms will be accompanied by annotated drawing images. It will be available in Latin and English and Latin	~ 6500	N/A	It will be a hierarchical structure. General divisions on major anatomical systems or regions of the body, specific regions or structures within each organ	No	Yes		It will be developed and curated by a expert panel of mouse morphologists

## Alignment of mouse and human anatomy ontologies and its application

Alignment of mouse and human anatomy ontologies facilitates the integration of mouse and human phenotype/genotype data and promotes the translation of basic research discoveries into clinical settings (Bodenreider et al. 2005). Such alignment is critical when integrating phenotype data from humans and mice in search of disease-causing genes (Boudellioua et al. 2017; Hoehndorf et al. 2011, 2013, 2015, 2011; Mungall et al. 2010; Robinson and Webber 2014; Schofield et al. 2010). However, the modelling decisions for the FMA were radically different from those in MA (Hayamizu et al. 2005a), reflecting that it was designed for different purposes, and the FMA contains a particularly rich set of relations, multiple axes of classification and concepts such as boundaries, landmarks, lines, voids and spatial relations, particularly relevant for human clinical imaging and surgical applications, for example. Both are excellent at what they do, but they were designed for different purposes. Some work has been done with anatomy ontology matching with terms from UMLS (https://www.nlm.nih.gov/research/umls), a widely used terminology that is not strictly an ontology, with a degree of success allowing for cross-species searching for example but not complex computation.

Under the Mouse–Human Anatomy Project (MHAP), the MA ontology and the human anatomical terms included in the National Cancer Institute (NCI) Thesaurus (Coronado et al. 2004) were compared. In the preliminary identification of equivalent terms using a combination of automated and manual curation approaches approximately 830 matching pairs were identified (Hayamizu et al. 2012). Afterwards, the harmonization of both ontologies by addition of terms represented only in one ontology, changes in the hierarchy, and augmented synonymy, reached 1634 matching terms, meaning that approximately half of the terms do not match and are specific to one of the two ontologies (Hayamizu et al. 2012). An overall comparison of automatically mappable terms (Bioportal; accessed 29.5.23) shows that while 74,000 of 105,000 FMA classes can be mapped lexically and organisationally to MA, this includes a very large number of one-to-many mappings (FMA to MA), contrasting with the 1634 manually matched terms above. This is due to the inclusion of concepts such as clustered anatomical structures, relations and subregions and the high degree of granularity of the FMA. For example there are 15 terms in FMA mapped onto one term, soleus, in the mouse. Only a very limited number of the species-specific terms identified represented real anatomical differences between the two species, and there were consequences of decisions made regarding the views and resources used to build each ontology. The MA ontology was based on major sources and expertise, including mouse atlases as well as anatomy and histology text resources. Once the list of terms was generated, each term was confirmed to represent a real mouse anatomical structure. However, MA ontology developers found that this confirmation was sometimes ambiguous, since numerous structures described in anatomy and histology textbooks do not have unambiguous evidence of their existence in the mouse, and then consequently were not included in the ontology (Hayamizu et al. 2005a).

There are additional complications in mappings from human to mouse, sometimes in the “views” of the topology, such as where concepts have been created to reflect clusters or features of more specific structures. In other cases, there are real debates about the way to describe structures and others where the topology is quite distinct across species. For example, in the mouse the prostate has identifiable anterior, dorsal, lateral, and ventral lobes (Ruberte et al. 2017); the dorsal and lateral being viewed in combination as the dorsolateral prostate. The human gland is much less easily differentiated into lobes but in the NCI thesaurus anatomy the lateral, medial, and posterior lobes are defined, and an additional term used; “overlapping zones”. Originally the human prostate was believed to contain five lobes, but these are only recognizable during development where it originates from five pairs of epithelial buds. The human prostate is unilobular but contains three identifiable zones: central, transitional and peripheral. The central zone surrounds the ejaculatory duct, the transitional zone surrounds the urethra, and the peripheral zone makes up most of the prostate, lying against the rectal wall on the dorsal surface of the gland (Ittmann 2018). Given the profound structural differences between the human and mouse prostates the question must also be raised as to whether a lobe-specific mapping between the species, as is often attempted, is biologically justified. It is, however, possible to map mouse and human at a broader concept level and simply map the human to mouse prostate, and that complete collection of lobes/zones from each species. This is a useful mapping approach but only reflects a ground truth when made at this higher organizational level. The MA ontology and NCI thesaurus both contain broader concepts prostate gland and prostate gland lobe, and these allow accurate mappings at a biologically meaningful level of granularity.

Breaking down the anatomical barriers between human and mouse anatomy, indeed between these and other metazoan anatomies, is an important part of data integration, particularly for functional genomics and, as mentioned above, discovery of human disease genes using model organism phenotypes. Given the problems of anatomy ontology mapping a cross-species anatomy ontology was developed—Uberon. The problems with data integration across species become more difficult with evolutionary distance and where the matching of human to model organism anatomies is desired to “de-silo” data, particularly phenotype data, mappings can only be done at a low level of granularity. Such a mapping of evolutionarily homologous structures and anatomical concepts was achieved with the Uberon ontology (Mungall et al. 2012; Haendel et al. 2014). While not as granular as MA, Uberon has become a de facto standard for expressing anatomical location in gene expression studies, and also through equivalence axioms facilitating cross-species phenotype similarity assessments using the major metazoan phenotype ontologies, for example MP (https://www.ebi.ac.uk/ols/ontologies/mp) and HPO (https://hpo.jax.org) (Robinson and Webber 2014; Gkoutos et al. 2017). As well as the incompleteness issues, Uberon has complex axes of classification and contains mixed semantic types. Detailed anatomical annotation of images, and high resolution phenotype capture were not applications for which Uberon was designed.

The use of anatomy ontologies, and specifically the MA ontology goes further than axiomatization and they have proved extremely useful in collecting and coding data for subsequent computational analysis. The collection of histopathology data at scale is complicated by the inability to compute on existing terminologies and the limited scope of existing terminologies when attempting to cover the entire domain of pathology in the mouse. Such terminologies would been many hundreds of thousands of concepts were they to pre-compose all the possible locations and types of lesions. This problem is solved by using an anatomy ontology, in this case MA, in combination with a foundational pathology ontology (MPATH) (Schofield et al. 2013) to post-compositionally create specific terms as required (Alghamdi et al. 2019). This approach has been applied for the high throughput pathology studies by the International IMPC (Elmore et al. 2018) and a large-scale study on the pathology of aging laboratory mice (Sundberg et al. 2011) using a dedicated data capture system, MODIS (Sundberg et al. 2008).

## Conclusions

The ability to relate and map anatomical structures between mice and humans is a critical part of using laboratory mice for the discovery of disease genes and pathological mechanisms. An accurate and granular formal terminology or ontology is essential for these activities, and permits not only accurate data capture, but also computational approaches to cross-species phenotyping. Such terminologies need to be grounded in biological truth and mappings made using knowledge-driven criteria. The problems mapping high resolution anatomical data from humans to mice and vice versa are, however, impacted by a major difference in the completeness of the two species’ anatomical descriptions. It is currently not possible to represent mouse anatomy with the precision available to FMA using the MA, and indeed the MA was not designed for the detailed representation required to annotate and interpret high resolution imaging and modern anatomical techniques, or the small variations seen between species and genetic backgrounds.

While it is not the aim of this commentary to propose a new anatomy ontology for the adult mouse, we have discussed how advances in imaging particularly have generated a requirement for more granular terminologies, the model for which is the TA or the NAV. Such knowledgebase level terminologies themselves have limited use computationally and we would strongly support establishment of a close relationship with MA curation to include new recommendations in the existing, gold-standard ontology as appropriate.

The study of human anatomy has a long history, mainly motivated by surgery and therapeutics. As we discuss above, it is currently more detailed and complete than mouse anatomy, so work to increase the accuracy and coverage of mouse anatomy will greatly assist in making accurate and meaningful mappings between the two species. The development of new imaging technologies, such as MRI, CT, SPECT, PET, two photon tomography (STP) (Ragan et al. 2012), light sheet microscopy (Stelzer et al. 2021) and experimental procedures such as CLARITY, 3DISCO and CUBIC (Ueda et al. 2020) have not only improved our knowledge of mouse anatomy but, in a sense, have turned anatomical investigations inside out. Traditionally human anatomy proceeded from the outside-in. Contemporary investigations can increasingly proceed from the inside-out (Standring 2016) and we are entering a new phase in the description and understanding of anatomy, where mouse anatomical studies may inform those in humans rather than vice versa.

Added to this is the increasing awareness of anatomically definable functional units which do not have morphologically explicit physical boundaries and structures at a macro-scale, yet can be demonstrated through high resolution molecular, imaging and functional techniques, has changed some of our concepts of what anatomy actually is, for example the brain connectome and the identification of the angiome (D'Angelo and Jirsa 2022; Taylor and Palmer 1987).

Integration of cellular and tissue anatomy is a topic we have not addressed in this commentary, but multiscale integration of anatomy is crucial to the generation of the virtual physiome (Hoekstra et al. 2018; Kokash and de Bono 2021) and much work is proceeding to make this computationally coherent in support of modelling physiological processes, which in turn allows for comparison of abnormal processes between species.

Finally, the need for training in these rapidly developing fields will be important in implementing the advantages gained from improved comparative anatomy between mice and humans, and training courses aimed at a new generation of investigators are under development in the community (Ruberte et al. 2020).
